# The Impact of Socioeconomic Factors on Cognitive Ability in Community-Dwelling Older Adults: Mediating Effect of Social Participation and Social Support

**DOI:** 10.3390/healthcare13050551

**Published:** 2025-03-04

**Authors:** Yilin Zheng, Yu Zhang, Mingzhu Ye, Tingting Wang, Huining Guo, Guohua Zheng

**Affiliations:** 1 Shanghai Institute for Global City, Shanghai Normal University, Shanghai 200234, China; yilinzheng@shnu.edu.cn; 2 School of Nursing and Health Management, Shanghai University of Medicine & Health Sciences, Shanghai 201318, China

**Keywords:** socioeconomic factors, cognitive ability, older adults, mediating effect, social participation, social support

## Abstract

Background and Purpose: Previous studies have shown that socioeconomic status influences cognitive health in adults. Therefore, it is important for the development of healthy aging policies to further investigate the effect of specific socioeconomic factors on cognitive function in older people and the possible mechanism. In this study, three specific socioeconomic factors (i.e., income, occupation, and education) were used as independent variables, and social support and social participation were used as the parallel or serial mediating variables to investigate the effect on cognitive function in community-dwelling older adults and the specific pathway of influence. Methods: A cross-sectional study was conducted in the Pudong New District of Shanghai, China. A total of 970 community-dwelling older adults aged over 60 years old who had lived in their current location for more than 5 years were enrolled. Socioeconomic factors in older adults, including income, education level, and occupation before retirement, were investigated, and their cognitive function and social support and social participation levels were measured using the MoCA, MSPSS, and the quantity of participation in social activities, respectively. Covariates, including lifestyle, health status, sleep quality, and nutritional status, were assessed using a self-designed questionnaire, the PSQI, and the MNA-SF scale. Omnibus mediation effect analysis was adopted to examine the mediation effect, and the mediation analysis was performed using the SPSS PROCESS program. Results: Community-dwelling older adults with higher income, more complex occupation, or higher education level had a better cognitive function, with β_medium income_ = 1.949 and β_high income_ = 3.799 compared to low-income level (all *p* < 0.001), β_medium occupational complexity_ = 1.262 and β_high occupational complexity_ = 1.574 compared to low occupational complexity level (all *p* < 0.01), and β_medium education_ = 1.814 and β_high education_ = 1.511 compared to low education level (all *p* < 0.001). Social participation significantly mediated the above relationship (all *p* < 0.001); the relative indirect effect of medium and high income through social participation was respectively β_medium income_ = 0.356 and β_high income_ = 0.777 compared to low income, accounting for 18.36% and 20.45% of the total effect; the relative indirect effect (β) of medium and high occupational complexity compared to low level of occupational complexity was 0.358 and 0.561, accounting for 28.36% and 35.64% of the total effect; while the relative indirect effect (β) of medium and high education compared to low education level was 0.311 and 0.562, with 17.14% and 39.19% of the total effect. Social support significantly mediated the relationship of income and education with cognitive function (all *p* < 0.001), with the indirect effect (β) of medium and high levels of income or education compared to their low levels being 0.132 and 0.160, or 0.096 and 0.156, respectively, accounting for 4.21% and 6.77%, or 5.29% and 10.32%, of their total effects. Serial mediation analysis showed that income and education significantly affected social participation through social support and subsequently cognitive function (all *p* < 0.01), with the relative serial indirect effects (β) of medium and high levels of income or education compared to their low levels being 0.065 and 0.078, or 0.043 and 0.070, respectively, accounting for 3.3% and 2.0%, or and 2.4–4.6% of their total effects. Conclusions: This study demonstrates that social support and social participation independently and cumulatively mediate the relationship between socioeconomic conditions and cognitive function in community-dwelling older adults. Therefore, improving the social support systems and encouraging older adults to actively participate in social activities may be beneficial in preventing or improving cognitive decline in community-dwelling older adults. The findings also provide new insights for the future improvement of cognitive function in community-dwelling older adults in the future.

## 1. Introduction

With the increase in global life expectancy, the prevalence of dementia or cognitive dysfunction is rising rapidly and is becoming a significant public health problem as a major cause of disability and mortality in the elderly population [1]. According to the 7th census, China has the largest elderly population in the world, with 264 million people over 60 years of age, or 18.7% of the total population [2]. Cognitive impairment has become one of the most common mental disorders among older adults in China, with an estimated prevalence of 20.4% [3]. Delaying or preventing cognitive decline is, therefore, a critical public health issue in an aging society.

Cognitive function is a key determinant of independence and quality of life in older adults, and healthy cognitive function enables older people to maintain social connections and independent functioning [4,5]. However, cognitive aging is inevitable, a process of gradual, progressive cognitive decline that occurs with age [6]. In addition, cognitive aging is complex and influenced by genetics, environment, and lifestyle. Genetic factors cannot be controlled, but many environmental factors and lifestyle factors can be modified or managed in early and mid-life experiences and are associated with cognitive health in later life [7,8].

### 1.1. Socioeconomic Factors and Cognition

Social determinants of health influence the incidence and prevalence of disease and health inequalities and are reported to account for 30–55% of health outcomes, exceeding even the contribution of medical factors [9]. Social determinants of health are generally defined as the environmental conditions in which individuals are born, live, learn, work, play, and worship, which influence a wide range of health outcomes [10]. A growing body of evidence has shown that the socioeconomic status of social determinants of health is a fundamental determinant of many health outcomes and can influence health outcomes through multiple pathways, including lifestyle and behaviors, access to health care, environmental exposures, physiological processes, and social–cultural and psychological pathways [11,12]. It is also an important factor influencing the cognitive health of older adults, with education, income, and occupational experience playing a key role [13,14]. A cohort study with nearly 30 years of follow-up (from 1985 to 2012) found that socioeconomic disadvantage, including low education and occupational position in midlife, was significantly associated with poorer cognitive function in later life [15]. Another 12-year longitudinal study of Mexican-origin adults also found that the trajectories of socioeconomic levels (i.e., per capita income, economic stress, educational attainment) were robustly associated with cognitive function and that higher initial levels and greater increases in socioeconomic resources had protective associations with cognitive function [16]. It is widely accepted that some of the effects of socioeconomic conditions on cognition are related to lifestyle differences, as individuals with lower socioeconomic status tend to have poorer diet quality and more risky health behaviors [17]. Additionally, low socioeconomic status, such as lower levels of education or occupational complexity, may also directly affect cognition through reduced cognitive reserve [18]. The exact mechanism is unknown, but it may play a key role in the pathway by which other social factors mediate cognitive function.

### 1.2. The Mediating Role of Social Participation and Social Support

Social participation, defined as engaging in activities that involve interacting with others in community life [19], plays a crucial role in the health and well-being of older adults [20]. A growing body of research has shown that positive participation in social activities is helpful in maintaining cognitive function or mitigating cognitive decline in middle-aged and older adults [21,22,23,24], while poor social participation was significantly associated with an increased risk of dementia-related conditions [9].

Social support, which is an individual’s social network of communication with others, is another important social factor [25]. Social support can be divided into four main functions, emotional, informational, friendship, and instrumental support, and is recognized as a protective factor against negative life experiences [26]. The positive impact of social support on health is dominated by continuous interaction and mutual influence with the individual’s social context, and perceived social support is beneficial in reducing the individual’s negative reactions to stressful experiences, leading to a reduction in the inflammatory response and in turn affecting cognitive function [27,28]. Longitudinal studies have also shown that the perceived social support from family members or friends can positively promote cognitive health in older adults [29,30].

Although socioeconomic factors, such as low income, unemployment, and low educational attainment, were negatively associated with cognitive function, positive participation in social activities and better social support may increase mental stimulation, synaptic density, and neuronal growth, promote social integration, and improve one’s social capital and healthy behaviors, which in turn may promote better cognition [31,32,33]. It is therefore possible that they mediate the effect of socioeconomic factors on cognitive function in older adults. However, no study has clarified the role of social participation and/or social support in the relationship between socioeconomic factors and cognitive function among community-dwelling older adults.

### 1.3. The Conceptual Model of Present Study

**Figure 1:**
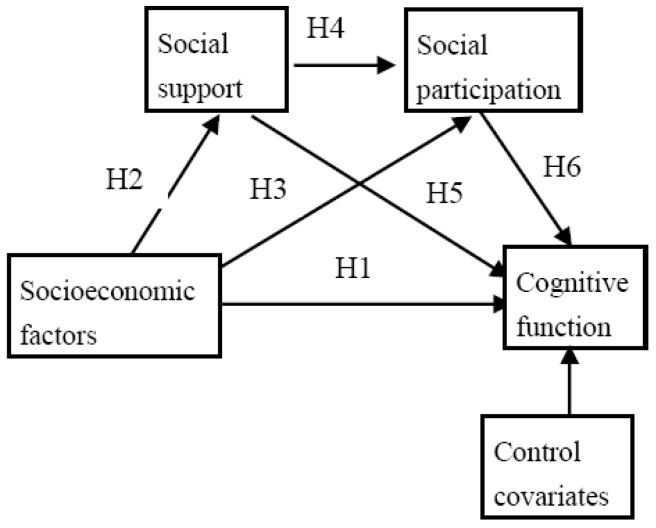
The theoretical framework of the study.

In the current study of cognitive function, socioeconomic factors, social participation, and social support are considered as parallel or serial factors that independently or interactively affect cognitive function in the community-dwelling elderly population. The aim of this study was to explore how and under what behavioral conditions socioeconomic factors affect cognitive function in community-dwelling older adults, and to inspire young adults to change their behaviors to protect their cognitive function in later life, by investigating the parallel and serial mediation models (Figure 1).

In terms of parallel mediation, the following main hypotheses were examined:

1. Socioeconomic conditions (income, occupation, and education) are directly associated with cognitive function (i.e., high levels of socioeconomic conditions are directly positively related to high levels of cognitive function in community-dwelling older adults (H1)).
2. Social support mediates the relationship between socioeconomic factors and cognitive function (i.e., high levels of socioeconomic conditions are significantly associated with high levels of social support (H2), which are significantly associated with high levels of cognitive function (H5)).
3. Social participation mediates the relationship between socioeconomic factors and cognitive function (i.e., high levels of socioeconomic conditions are significantly associated with high levels of social participation (H3), which are significantly associated with high levels of cognitive function (H6)).

In this mediation model, it is hypothesized that two mediating variables independently influence the association of socioeconomic factors with cognitive function. For the indirect effects of socioeconomic factors on cognitive function, higher levels of socioeconomic conditions are associated with higher levels of social support or social participation, which in turn are associated with higher levels of cognitive function.

In terms of serial mediation, two mediating variables are hypothesized to act sequentially:

4. Social support mediates the relationship between socioeconomic conditions and cognitive function, which in turn is mediated by social participation (i.e., high levels of socioeconomic conditions lead to high levels of social support, which in turn lead to high levels of social participation, and ultimately lead to high levels of cognitive function (path from H2, H4, to H6)).

## 2. Methods

### 2.1. Participants and Procedures

A total of 1000 community-dwelling older adults were recruited from four residential communities in Pudong New Area, Shanghai City, China, using online and offline convenience sampling methods including internet recruitment, posters, leaflets, and on-site recruitment stations. They were eligible to participate in the study if they were aged 60 years or older, had lived in their current location for more than 5 years, and gave informed consent. After obtaining informed consent from community-dwelling older adults, a face-to-face interview was conducted to collect the variable information. Data collection was completed between 1 December 2022 and 1 May 2023. A total of 979 eligible older adults were identified excluding 21 participants who refused to cooperate with the completion of the study. The procedures in this study adhered to the Declaration of Helsinki and were approved by the Ethics Committee of the author’s institution (No. 2022-ZGH-013). A post hoc power analysis was conducted using G*power 3.1 to assess the current effective sample size for correlation analysis. With a total sample size of 925 participants, a significant level of α = 0.05, and an effect size of r = 0.1, the statistical power reached 0.92 [34].

### 2.2. Measures

#### 2.2.1. Cognitive Function

Participants’ cognitive function was assessed using the Montreal Cognitive Assessment (MoCA) scale (Beijing version). The MoCA scale consists of eight sub-dimensions of cognitive function, each of which assesses the visuospatial/executive function, naming, memory, attention, verbal fluency, abstraction, delayed recall, and orientation, respectively. The maximum score on the MoCA scale is 30 points, with higher scores indicating better cognitive functioning [35].

#### 2.2.2. Socioeconomic Factors

The socioeconomic factors in this study mainly included three socioeconomic variables: education, income, and occupational experience before retirement. In order to reflect the lifetime exposure of community-dwelling older adults to socioeconomic conditions, the income, education, and occupational exposure of each participant in this study were identified as the retirement pension, education and training experience, and occupational complexity before retirement. Due to the well-established pension insurance system in Shanghai, China, most of the community-dwelling older adults in Shanghai have a fixed retirement pension, and the retirement pension is generally calculated on the basis of the individual’s social pension contributions paid during his or her working life, and the individual’s pension contributions are positively proportional to his or her wage income. For this reason, the retirement pension can be used as an indicator for the assessment of the income level of older adults before their retirement. According to the average retirement pension and the basic living expenses of the general inhabitants in Shanghai City, the income level of the participants was divided into 3 categories: less than 2000 CNY/month as low level, 2000–6000 CNY/month as medium level, and more than 6000 CNY/month as high level. The educational level in this study was assessed by a combined score of years of formal education and vocational or training courses, where each year of primary, secondary, and high school was assigned 1 point if successfully completed, and the vocational or training course was assigned 0.5 points for each 6-month course. The educational level of the participants was categorized as low, medium, or high according to the total education scores, using the tertile principle. Occupational exposures included the type and years of work in adulthood occupations before retirement, and work activities were categorized into six levels according to the type of work: low-skilled manual work, skilled manual work, skilled non-manual or technical work, professional work, and highly intellectual work. Scores for occupational complexity were calculated as the product of the level and the years of work activity in adulthood. Occupational levels were divided into three classes according to the total occupational scores, namely, low, medium, and high, based on the tertile principle [36].

#### 2.2.3. Social Participation and Social Support

Social participation was defined as recent participation in any social activity. It includes five types of activities and is scored on a 4-point scale, with “not participating” scoring 1, “not regularly (about once a month)” scoring 2, “sometimes (about every week)” scoring 3, “often (almost daily)” scoring 4; higher scores indicate higher levels of participation [37].

Social support was assessed using the Multidimensional Scale of Perceived Social Support (MSPSS), which consists of 12 items assessing perceptions of social support from family, friends, and other significant sources, and each item is scored from 1 to 7 according to the degree of agreement (very strongly disagree = 1, strongly disagree = 2, mildly disagree = 3, neutral = 4, mildly agree = 5, strongly agree = 6, very strongly agree = 7), with higher total scores indicating greater social support [38].

#### 2.2.4. Covariates

Participants’ demographic characteristics (e.g., age, sex, height, weight, marital status, place of residence), lifestyle (e.g., smoking, drinking), and health status (e.g., chronic diseases) were assessed using a self-designed questionnaire. Body mass index (BMI) was calculated as body weight (kg) divided by the square of height (m). Sleep quality was assessed using the Chinese version of the Pittsburgh Sleep Quality Index (PSQI) [39]. Nutritional status was assessed using the Chinese version of the Mini Nutritional Assessment Short Form (MNA-SF) [40]. The definition and assignment of the variables are shown in Table 1.

### 2.3. Statistical Analysis

**Figure 2:**
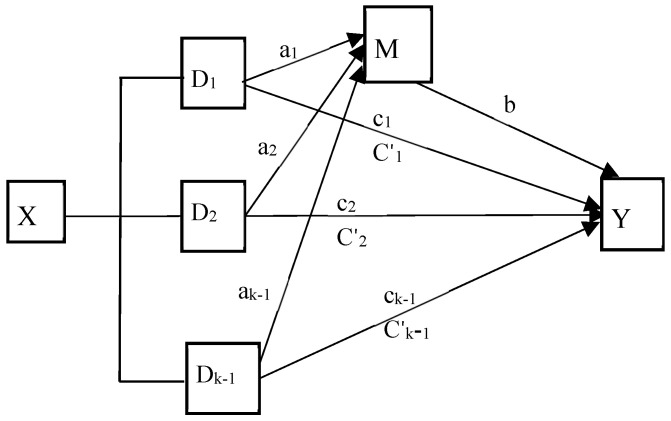
The mediation model with multi-categorical independent variable (X: independent variable; Y: dependent variable; M: mediation variable; a_i_: the relative effects of the other k − 1 levels, respectively, relative to the k level, on mediation variable; b: the association between mediation variable and dependent variable; c_i_: relative total effect of the k − 1 levels, respectively, relative to the k level, on dependent variable; C′_i_: relative direct effects of the k − 1 levels, respectively, relative to the k level, on dependent variable after controlling for mediation variable. i = 1, 2, … k − 1).

IBM SPSS Statistics software (version 25) was used for all the statistical analyses, and the difference was considered statistically significant with a 95% confidence interval (95% CI) or *p* < 0.05 on both sides. Missing data were imputed using Markov Chain Monte Carlo (MCMC) multiple imputation. Descriptive statistics were expressed as the means and standard deviations (SDs) for the continuous variables and frequencies and percentages for categorical variables. Independent samples *t*-test or F-test was used to identify covariates that may be related to cognitive function by comparing the difference in cognitive function between different characteristics/levels of covariates. Pearson correlation coefficients were used to assess the relationship between cognitive function and social participation and social support. The SPSS macro PROCESS 4.1 plug-in was used to conduct a mediation analysis [41]. Model 4 and Model 6 were used to examine the parallel or serial mediation effect of social participation and social support between socioeconomic factors and cognitive function, respectively. Given that the three independent variables were multi-categorical variables, the omnibus mediation effect analysis based on the research findings of Preacher and Hayes was performed before the mediation effect analysis [42]. The k dummy variables were set for each multi-categorical independent variable, and k-1 relative direct and relative indirect effects relative to the reference level were estimated (Figure 2). If the omnibus effect was not significant, it means that the potential mediation effect did not exist and the medication effect analysis was not performed. k dummy variables were set for each multi-categorical independent variable, and the k-1 direct and indirect effects were estimated as the k-1 relative mediation effect. The bootstrap method with a sample size of 5000 was used to examine the mediation effect. All tests were within the 95% confidence interval (95% CI). If the interval of 95% CI did not include zero, it indicated that the mediating or moderating effect was significant at the 0.05 level.

## 3. Results

### 3.1. Analysis of Common Method Bias

The Harman single-factor test with the untwisted principal component factor mothed was used to analyze the potential common method bias for all variables in the current data. The results showed that the first factor explained 14.2% of the variance, indicating that there was no obvious common method bias in the current research data.

### 3.2. Descriptive Statistics and Correlation Analysis for the Study Variables

The samples consisted of 979 community-dwelling older adults aged over 60 years old recruited from the residential area of Pudong district in Shanghai, China. Table 2 shows the comparison of cognitive function in different characteristics of demographic, independent, and covariate variables among participants. There were significant differences in MoCA scores between different levels of participants’ income, occupation, and education (all *p* < 0.01). Age, marital status, smoking, drinking, nutritional status, and sleep quality significantly affected the participants’ cognitive function (all *p* < 0.05). Correlational analysis showed that social participation and social support were positively correlated with cognitive function (Table 3).

### 3.3. Mediation Effect Analysis

#### 3.3.1. Omnibus Mediation Effect Analysis

The analysis of the omnibus effect after controlling for the older adult’s age, marital status, smoking, drinking, nutritional status, and sleep quality is shown in Table 4. When cognitive function was taken as the dependent variable and socioeconomic factors (income, occupation, and education) as the independent variables, regardless of whether social participation or social support was taken as the mediating variable, all of the omnibus total effect tests and the omnibus direct effect tests were significant (all *p* < 0.05), indicating that at least one of the relative total effects and relative direct effects were not equal to 0. The bootstrap 95% CI of all the omnibus mediation effect tests did not include 0, indicating that further relative mediation effect analysis was needed.

#### 3.3.2. Relative Mediation Effect of Social Participation or Social Support

**Figure 3:**
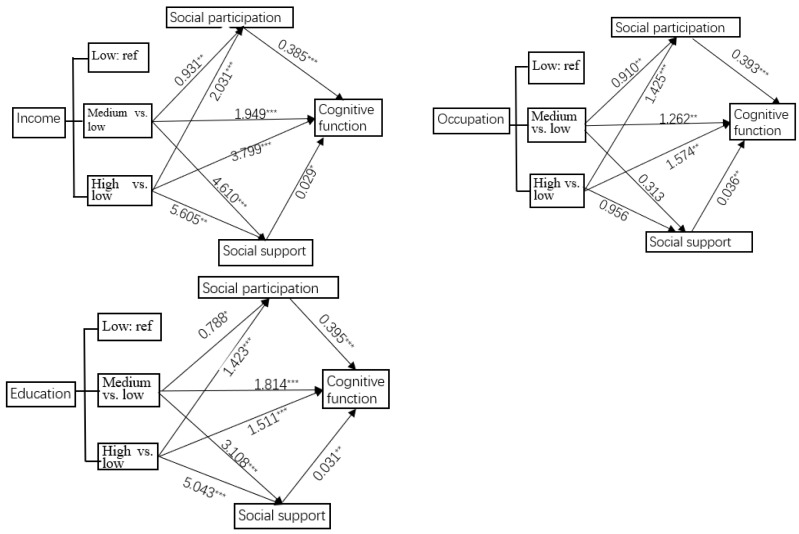
Social participation or social support plays a mediating role between socioeconomic conditions (e.g., income, occupation, and education) and cognitive function. The results showed that social participation and social support can positively mediate the relationship between socioeconomic factors and cognitive function. * *p* < 0.05; ** *p* < 0.01; *** *p* < 0.001.

Based on the result of the omnibus effect analysis, the relative total effect test and the relative mediation effect test were carried out (Figure 3 and Supplementary Table S1). As shown in Table 5, in terms of mediation effects through social participation, there is a significant relative mediation effect of income, occupation, and education level on cognitive function through social participation. Compared with the low-income group (retirement pension < 2000 CNY/month), older adults with medium or high income were more likely to improve their cognitive function through social participation. The relative mediation effect accounted for 18.36% (a_i_b = 0.358, 95% CI: 0.110~0.614) and 20.45% (a_i_b = 0.777, 95% CI: 0.349~1.222) of the relative total effect, respectively, indicating that 18.36% and 20.45% of the improvement in cognitive function among middle- and high-income older adults was mediated by social participation. Compared to those with low occupational experience (occupational scores < 53), older adults with more complex occupational experience could improve their cognitive function through more social participation. Among the older adults with medium or high levels of occupational experience, 28.36% (a_i_b = 0.358, 95% CI: 0.123~0.614) and 35.64% (a_i_b = 0.561, 95% CI: 0.299~0.851) of the improvement in cognitive function was mediated by social participation, and the relative mediation effect increased with the level of occupational experience promoted. Older adults with higher levels of education had better cognitive function than those with low levels of education, partly mediated by their social participation. The proportion of relative mediation effect was 17.14% (a_i_b = 0.311, 95% CI: 0.058~0.572) for medium education and 39.19% (a_i_b = 0.562, 95% CI: 0.294~0.851) for high education.

For the social support mediation model, there was a significant relative mediation effect of income and education level on cognitive function through social support. Specifically, compared with the low-income group, the older adults with medium or high income had better cognitive function, in which the proportion of the relative mediation effect of social support was 6.77% (a_i_b = 0.132, 95% CI: 0.019~0.282) and 4.21% (a_i_b = 0.160, 95% CI: 0.017~0.372), respectively; compared with the low educational level, the older adults with medium or high educational level were more likely to improve their cognitive function by social support, and the proportion of relative mediation effect was 5.29% (a_i_b = 0.096, 95% CI: 0.013~0.214) and 10.32% (a_i_b = 0.156, 95% CI: 0.035~0.318), respectively.

#### 3.3.3. Relative Seral Mediating Effect of Social Support and Social Participation

**Figure 4:**
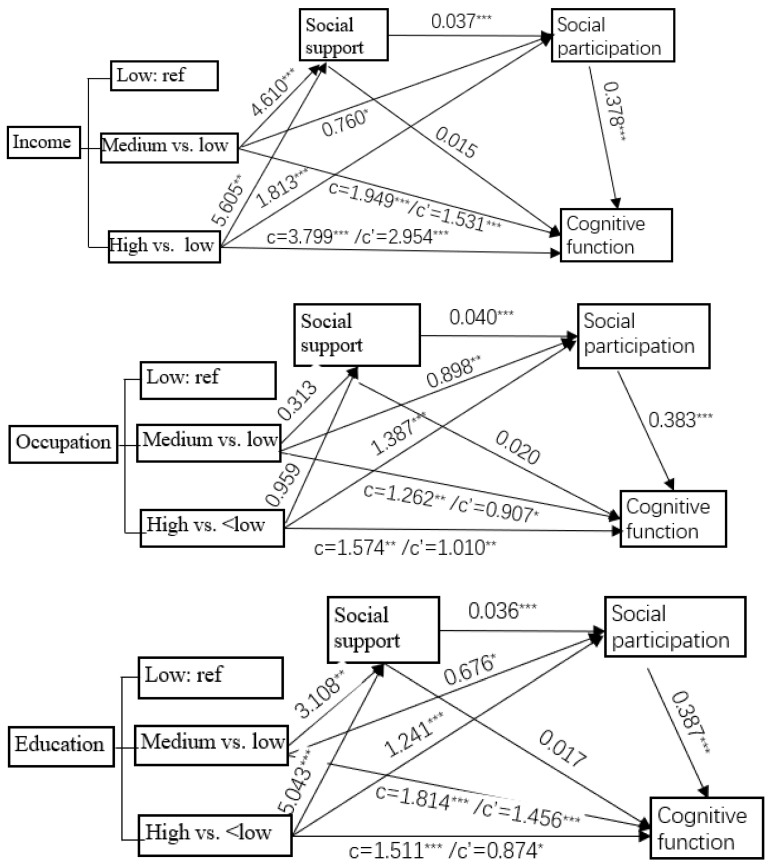
Examined serial mediation model of social support and social participation on socioeconomic factors (income, occupation, and education) and cognitive function in community-dwelling older adults. * *p* < 0.05; ** *p* < 0.01; *** *p* < 0.001.

The examined serial mediation effect of social support and social participation on socioeconomic factors on cognitive function is shown in Figure 4 and Supplementary Table S2. Table 6 shows the relative direct effect of each independent and mediating variable in the serial mediation model after controlling for the older adult’s age, marital status, nutritional status, and sleep quality. In the model of the relationship between income or education and cognitive function, all of the direct effects were significant except for the social support for cognitive function. In the model of the relationship between occupation and cognitive function, medium or high occupation was found to have no significant direct effect on social support compared with low occupation level; social support was found to have no significant direct effect on cognitive function.

Table 7 shows the indirect effect of socioeconomic conditions (e.g., income, occupation, education), social support, social participation, and cognitive function after controlling for the older adult’s age, marital status, smoking, drinking, nutritional status, and sleep quality. As shown in Table 7, in the model from income to cognitive function, compared with the low-income level, the medium- and high-income level affected cognitive function through social participation by β = 0.287 (95% CI: 0.042~0.539) and 0.685 (95% CI: 0.261~1.136), accounting for 14.7% and 18.0% of the total effect, respectively, and through social support and then social participation (i.e., serial mediation effect) (β = 0.065, 95% CI: 0.025~0.115; β = 0.078, 95% CI: 0.023~0.152), explaining 3.3% and 2.0% of the total effect, respectively. But the mediating effect through social support was not significant. These results suggest that the indirect effect of medium or high income on cognitive function through social support is completely mediated by social participation.

In the model from occupation to cognitive function, compared to low levels of occupational complexity, social participation mediated the association between medium and high levels of occupational complexity and cognitive function with β = 0.344 (95% CI: 0.117~0.591) and β = 0.531 (95% CI: 0.277~0.824), accounting for 27.3% and 33.7% of the total effect, respectively. There was no significant mediation effect through social support and no serial mediation effect (first through social support, then through social participation) on the association between medium and high levels of occupational complexity and cognitive function.

In the model from education to cognitive function, compared with low educational level, medium and high educational level affected cognitive function through social participation with β = 0.262 (95% CI: 0.020~0.515) and 0.480 (95% CI: 0.224~0.761), explaining 14.4% and 31.8% of the total effect and through serial mediation (first through social support, then through social participation) with β = 0.065 (95% CI: 0.025~0.115) and β = 0.078 (95% CI: 0.023~0.152), explaining 2.4% and 4.6% of the total effect, respectively; no significant mediation effect was found through social support.

## 4. Discussion

This study aimed to examine the interplay between socioeconomic conditions, social participation, and social support on cognitive function by investigating the parallel and serial mediating role of social support and social participation in the relationship between socioeconomic factors and cognitive function. The current study revealed three important findings. First, an individual’s socioeconomic level, including income, education, and occupational exposure in adulthood, was positively associated with cognitive function. Second, social participation significantly mediated the association between socioeconomic conditions and cognitive function, and high levels of social participation may strengthen the effect of socioeconomic factors on cognitive function in community-dwelling older adults; social support significantly mediated the income and education on cognitive function, and social support positively strengthens the effect of income and education on cognitive function in community-dwelling older adults. Third, social participation completely mediated the effect of the socioeconomic factors (income and education) on cognitive function through social support (i.e., the indirect effect of income and education on cognitive function through social support is completely mediated by social participation).

This study confirmed that an individual’s socioeconomic conditions are positively associated with cognitive function; in other words, the higher the level of income, education, or occupational experience, the better the cognitive function of community-dwelling older people, which is in line with currently accepted opinion [43,44]. A growing body of research, using sophisticated behavioral and neuroimaging measures, has demonstrated the causal linkages between these socioeconomic factors and specific cognitive functions [45,46]. Higher socioeconomic conditions across life stages were independently and cumulatively associated with better neural or cognitive outcomes, as reflected by increased cortical thickness, grey matter volume, fractional anisotropy, and network segregation in adults [47,48]. The economic viewpoint suggests that individuals with greater economic resources were better able to purchase important products related to cognitive or brain development, such as nutritious foods, enriching learning opportunities, etc. [49]. The evidence shows that educational attainment has a positive effect on cognitive function, and that the number of years of formal education completed by individuals is positively associated with their cognitive function in adulthood and is predictive of a lower risk of dementia in later life, and that educational attainment influences cognitive function in later life primarily by contributing to individual differences in cognitive ability that emerge in early adulthood but persist into old age [50]. Adult work experience, as a general mentally stimulating activity, was found to be able to influence cognitive function in later life, and more complex adult work activities were associated with better cognitive function in later life [51,52]. The complex work activities also stimulate higher mental or intellectual demands related to specific cognitive processes [53,54].

Second, this study roughly validated the hypothesis that social participation and social support independently mediate the effect of socioeconomic conditions on cognitive function in community-dwelling older people. Specifically, when social participation was the mediating variable, it mediated about 18–20%, 28–35%, and 17–39% of the differences in cognitive function due to income inequality and educational and occupational gaps. Moreover, the mediating effect of social participation is stronger the greater the difference between levels of socioeconomic condition. Social participation, such as good and cordial relations with family, relatives, and friends, is the main source and the most important factor of life satisfaction and happiness of individuals, which are indirectly beneficial for cognitive function [55]. Although poor socioeconomic conditions can affect an individual’s physical, mental, and cognitive health by limiting access to needs and opportunities [56], positive social participation could at least partially offset these negative effects of socioeconomic inequalities [57]. Existing studies have shown that the socioeconomic gap can indeed have a significant effect on the physical and mental health of older people, but that this effect may be partly explained by the mediating effect of social participation [58,59]. Serval previous studies have shown that social participation could mediate several factors influence mental health of adults. For example, Zheng J et al. reported a partial mediating effect of social participation on the relationship between activities of daily living and anxiety in older adults [60]; Wu B et al. found that social participation could effectively mediate the effect of exercises on the mental health of urban older adults living alone [61]. This viewpoint is further supported by this study’s results.

When social support was the mediating variable, we found the weak mediating effect of social support on the association between income and education of socioeconomic conditions and cognitive function in community-dwelling older adults. More specifically, social support mediated about 4.2–6.8% and 5.3–10.3% of the disparity in cognitive function due to the income or education gap, with the larger the income or education gap, the higher the proportion of mediation; no significant mediating effect of social support was found for the association between the occupational complexity and cognitive function. Social support, as part of a social network of mutual help and obligation, is one of the active ingredients in the health benefits of being connected [62], and the proposed neural mechanism is related to enhancing ventral striatum and septal area activity and inhibiting parental care (e.g., amygdala), which is linked to downstream stress-related responses [63]. One study has reported that social support, including informational, instrumental, and financial support, is an effective buffer against the negative effects of poverty on mental health [64]. Another study also found significant differences in the cognitive benefits of receiving support from family or friends among older adults with different educational levels [65]. These findings were similar to the results of this study.

Finally, this study confirmed that social participation can mediate the role of income, education, and social support in promoting cognitive function in community-dwelling older adults. We found that high levels of income and education were associated with high levels of social support, which led to high levels of social participation and improved cognitive function. Social participation has been theorized to mitigate age-related declines in emotional functioning and may be more beneficial than social support in buffering against these declines [66]. However, when mediated by social participation, there was no significant effect of social support on cognitive function. These results suggest that the partial effects of high levels of income and education on cognitive function are due to high levels of social support and social participation, and that the effect of social support on cognitive function is entirely due to the mediation of social participation. Socioeconomic status, such as income and education levels, is an important cause of the unequal distribution of social support, and individuals with lower socioeconomic status have smaller social networks and less organizational involvement [67,68]. Therefore, socioeconomic level affects to some extent an individual’s access to social support, which in turn affects an individual’s social participation. Given that positive social support and social participation have a positive relationship with cognitive function, increasing these types of social connections may promote cognitive function in older adults, also offsetting the negative effect of low socioeconomic conditions on cognitive function [31]. Moreover, social participation may be more beneficial than social support in buffering this decline; social participation may optimize an individual’s social network, which facilitates only high-quality social interactions, and may be an important facet influencing well-being [66].

In terms of application, the findings of this study may be reflected in an individual’s ability to manage the cognitive aging process. Social support and social participation may be considered as behavioral tools to help older adults cope with cognitive changes resulting from socioeconomic inequality in adulthood, and social participation could be more effective in helping facilitate cognitive function. For community-dwelling older adults, improving their social support network may increase their opportunities to participate in community activities and may be helpful in promoting their cognitive health and offsetting the negative effects of socioeconomic inequality in their adulthood.

There are several limitations to this study. First, the cross-sectional design of this study limits the ability to draw conclusions about causality. Therefore, future research should strengthen these findings by using a longitudinal design. Second, this study investigated the social support and social participation of older adults in their later years. Therefore, the results may not account for the long-term effects of social support and social participation on cognitive function. Third, the information on socioeconomic factors is self-reported, so the possibility of misclassification bias may not be excluded. Finally, the information on the controlled confounders only reflects the conditions at the time of the investigation and not at the time of exposure, which could influence the results. Fourth, selection bias may be unavoidable due to convenience sampling. In addition, although the main confounders were controlled in this study, potential bias from unmeasured confounders cannot be avoided. Therefore, future studies with more robust sampling are needed. Despite the limitations mentioned above, our findings may have relevant public health implications. First, our findings suggest the importance of assessing socioeconomic conditions when investigating risk factors for cognitive impairment, even from a life course perspective. Second, as social support and social participation may effectively mediate the impacts of socioeconomic factors on cognitive function, improving social support networks or actively participating in social activities may be an effective intervention to prevent cognitive decline in older adults.

## 5. Conclusions

The results showed that socioeconomic factors can directly and indirectly affect the cognitive function in community-dwelling older adults, and that social support and social participation, alone or in combination, mediate the effect of socioeconomic conditions on cognitive function. These findings provide some insights into the potential pathways between socioeconomic conditions and cognitive function, as well as the protective role of social support and social participation for cognitive function in community-dwelling older adults, suggesting that policymakers should provide social support to maintain the social relationship network for the participation of community-dwelling older adults.

## Figures and Tables

**Table 1:** Variable definition and assignment.

Variables	Variable Definition and Assignment
Dependent variable	
Cognitive function	MoCA scores. Value range: 0–30 scores
Independent variables	
Income	Retire pension monthly. 1 = less than 2000 CNY/month; 2 = 2000–6000 CNY/month; 3 = more than 6000 CNY/month
Education	Educational scores. 1 = less than 6 scores; 2 = 6–10 scores; 3 = more than 10 scores.
Occupation	Occupational complexity scores. 1 = less than 53 scores; 2 = 53–90 scores; 3 = more than 90 scores
Mediating variables	
Social participation	Scores. Value range:5–20 scores
Social support	Scores. Value range: 12–84 scores
Covariates	
Age	1 = 60–65 years old; 2 = 66–75 years old; 3 = 71–75 years old; 4 = more than 75 years old
Sex	1 = male; 2 = female
Marital status	1 = married and living with a spouse; 2 = separated (widowed or divorced)
Sleep quality	PSQI scores: 1 = less than 5 scores; 2 = 5–10 scores; 3 = more than 10 scores
Smoking status	1 = current smoking; 2 = never or quit smoking
Drinking status	1 = current drink; 2 = never or occasional drinking
BMI (kg/m^2^)	1 = less than 24; 2 = more than or equal to 24
Nutrition status	MNA scores: 1 = less than 12 scores; 2 = more than and equal to 12
Co-morbidities	1 = none; 2 = one chronic disease; 3 = more than two diseases

**Table 2:** Comparison of cognitive function among the sample characteristics.

Categorical Measures		N (%)	MoCA (Scores) Mean (SD)	*p* Value
Income (CNY/month)	<2000	205 (20.9%)	18.06 ± 5.60	
	2000~6000	695 (71.0%)	20.31 ± 5.30	
	>6000	79 (8.1%)	22.40 ± 5.08	<0.001
Occupation (scores)	<53	325 (33.2%)	19.12 ± 5.86	
	53~90	336 (34.2%)	20.33 ± 5.29	
	>90	318 (32.5%)	20.57 ± 5.10	0.01
Education (scores)	<6	315 (32.2%)	18.50 ± 6.03	
	6~10	330 (33.7%)	20.85 ± 4.61	
	>10	331 (33.8%)	20.56 ± 5.40	<0.001
Sex	Male	413 (42.4%)	20.14 ± 5.40	
	Female	564 (57.6%)	19.91 ± 5.50	0.501
Age (years)	60~65	288 (29.4%)	21.33 ± 4.57	
	66~70	336 (34.3%)	20.48 ± 4.89	
	71~75	169 (17.3%)	19.99 ± 5.61	
	>75	185 (18.9%)	17.07 ± 6.44	<0.001
BMI (kg/m^2^)	<24	586 (59.9%)	19.96 ± 5.40	
	≥24	374 (38.2%)	20.05 ± 5.58	0.794
	Missing	19 (1.9%)		
Marital status	Married	848 (86.6%)	20.33 ± 5.27	
	Separated	131 (13.4%)	17.89 ± 6.13	<0.001
Smoking	Yes	177 (18.1%)	20.67 ± 4.85	
	No or quit	802 (81.9%)	19.86 ± 5.58	0.050
Drinking	Yes	154 (15.7%)	20.90 ± 4.71	
	No or quit	825 (84.3%)	19.84 ± 5.57	0.014
Co-morbidities	None	163 (16.6%)	19.90 ± 5.80	
	1	375 (38.3%)	20.09 ± 5.43	
	≥2	242 (24.7%)	19.39 ± 5.64	0.311
	Missing	199 (20.3%)		
Nutrition status (MNA, scores)	≤12	30 (6.1%)	19.32 ± 5.67	
	>12	465 (93.9%)	20.29 ± 5.35	0.011
Sleep quality (PSQI, scores)	<5	573 (58.5%)	20.63 ± 5.26	
	5~10	327 (33.4%)	19.38 ± 5.57	
	>10	79 (8.1%)	18.12 ± 5.72	<0.001

**Table 3:** Descriptive statistical results and correction analysis between dependent and moderating variables (Pearson correlation coefficient).

Variables	Mean (SD)	Cognitive Function (MoCA, Scores)	Social Participation (Scores)	Social Support (Scores)
Cognitive function (MoCA, scores)	20.1 (5.46)	1		
Social participation (scores)	11.3 (4.1)	0.350 ***	1	
Social support (scores)	59.1 (14.8)	0.139 ***	0.169 ***	1

*** *p* < 0.001.

**Table 4:** Omnibus effect analysis (F values).

Mediation Variable	Independent Variables	Dependent Variable (Cognitive Function)		
		Omnibus Total Effect	Omnibus Direct Effect	Bootstrap 95% CI
Social participation	Income	18.294 ***	12.759 ***	0.307~0.461
	Occupation	8.487 ***	4.088 **	0.313~0.471
	Education	11.120 ***	7.481 ***	0.316~0.472
Social support	Income	18.294 ***	16.301 ***	0.005~0.053
	Occupation	8.487 ***	8.258 ***	0.013~0.058
	Education	11.120 ***	9.594 ***	0.008~0.054

** *p* < 0.01, *** *p* < 0.001 after controlling age, marital status, smoking, drinking, nutrition status, and sleep quality. CI = confidence interval; the bootstrap 95% CI was for the omnibus mediation effect.

**Table 5:** Relative mediation effect of the mediation variables.

Mediation Variables	Socioeconomic Conditions	Cognitive Function			
		c_i_	a_i_b	Bootstrap 95% CI	|a_i_b/c_i_|
Social participation	Income (ref: <2000 CNY/month)				
	2000~6000	1.949 ***	0.358	0.110~0.614	18.36%
	>6000	3.799 ***	0.777	0.349~1.222	20.45%
	Occupation(ref: <53 scores)				
	53~90	1.262 **	0.358	0.123~0.614	28.36%
	>90	1.574 **	0.561	0.299~0.851	35.64%
	Education (ref: <6 scores)				
	6~10	1.814 ***	0.311	0.058~0.572	17.14%
	>10	1.511 ***	0.562	0.294~0.853	39.19%
Social support	Income (ref: <2000 CNY/month)				
	2000~6000	1.949 ***	0.132	0.019~0.282	6.77%
	>6000	3.799 ***	0.160	0.017~0.372	4.21%
	Occupation(ref: <53 scores)				
	53~90	1.262 **	0.011	−0.073~0.103	--
	>90	1.574 **	0.034	−0.042~0.132	--
	Education (ref: <6 scores)				
	6~10	1.814 ***	0.096	0.013~0.214	5.29%
	>10	1.511 ***	0.156	0.035~0.318	10.32%

Ref = reference category; c_i_ = the relative total effects of every category in categorical variables on the dependent variable; a_i_b = the quantity of relative mediation effect; |a_i_b/c_i_| = the proportion of relative mediation effect; CI = confidence interval. ** *p* < 0.01, *** *p* < 0.001 adjustment for age, marital status, smoking, drinking, nutrition status, and sleep quality.

**Table 6:** Relative direct effect of each variable in the serial mediation model.

Relative Direct Effects	β	S.E	t	P	95% CI of β	
					LLCI	ULCI
Income (refer = low income level)						
Medium income → social support	4.610	1.144	4.029	<0.001	2.365	6.855
Medium income → social participation	0.760	0.319	2.384	0.017	0.134	1.385
Medium income → cognitive function	1.531	0.394	3.887	<0.001	0.758	2.303
High income → social support	5.605	1.937	2.894	0.004	1.805	9.406
High income → social participation	1.813	0.537	3.373	0.001	0.758	2.868
High income → cognitive function	2.954	0.666	4.437	<0.001	1.647	4.261
Social support → social participation	0.037	0.009	4.175	<0.001	0.020	0.055
Social support → cognitive function	0.015	0.011	1.321	0.184	−0.007	0.036
Social participation → cognitive function	0.378	0.04	9.535	<0.001	0.300	0.455
Occupation (refer = low level of occupation complexity)						
Medium occupation → social support	0.313	1.123	0.278	0.781	−1.891	2.516
Medium occupation → social participation	0.898	0.307	2.928	0.003	0.296	1.499
Medium occupation → cognitive function	0.907	0.383	2.366	0.018	0.155	1.660
High occupation → social support	0.956	1.137	0.841	0.401	−1.276	3.188
High occupation → social participation	1.387	0.311	4.465	<0.001	0.777	1.997
High occupation → cognitive function	1.010	0.391	2.583	0.010	0.243	1.776
Social support → social participation	0.040	0.009	4.559	<0.001	0.023	0.057
Social support → cognitive function	0.020	0.011	1.841	0.066	−0.001	0.042
Social participation → cognitive function	0.383	0.040	9.562	<0.001	0.304	0.461
Education (refer = low educational level)						
Medium education → social support	3.108	1.138	2.732	0.006	0.875	5.341
Medium education → social participation	0.676	0.316	2.141	0.033	0.056	1.296
Medium education → cognitive function	1.456	0.392	3.713	<0.001	0.687	2.226
High education → social support	5.043	1.138	4.431	<0.001	2.810	7.276
High education → social participation	1.241	0.318	3.906	<0.001	0.618	1.865
High education → cognitive function	0.874	0.397	2.203	0.028	0.096	1.653
Social support → social participation	0.036	0.009	4.044	<0.001	0.019	0.053
Social support → cognitive function	0.017	0.011	1.529	0.127	−0.005	0.039
Social participation → cognitive function	0.387	0.040	9.704	<0.001	0.309	0.465

SE = standard error; β = regression coefficient; CI = confidence interval; LLCI = lower level confidence interval; ULCI = upper level confidence interval.

**Table 7:** Relative mediation effect of the serial mediation variables.

Socioeconomic Conditions	Mediation Path	Cognitive Function			
		c_i_	a_i_b	Bootstrap 95% CI of a_i_b	|a_i_b/c_i_|
Income (ref: low-income level)					
Medium level	Social support	1.949 ***	0.067	−0.032~0.193	---
	Social participation	1.949 ***	0.287	0.042~0.539	14.7%
	Social support → social participation	1.949 ***	0.065	0.025~0.115	3.3%
High level	Social support	3.799 ***	0.082	−0.038~0.243	---
	Social participation	3.799 ***	0.685	0.261~1.136	18.0%
	Social support → social participation	3.799 ***	0.078	0.023~0.152	2.0%
Occupation (ref: low level of occupation complexity)					
Medium level	Social support	1.262 **	0.006	−0.047~0.070	---
	Social participation	1.262 **	0.344	0.117~0.591	27.3%
	Social support → social participation	1.262 **	0.005	−0.030~0.044	---
High level	Social support	1.574 **	0.019	−0.028~0.090	---
	Social participation	1.574 **	0.531	0.277~0.824	33.7%
	Social support → social participation	1.574 **	0.015	−0.019~0.055	---
Education (ref: low educational level)					
Medium level	Social support	1.814 ***	0.053	−0.013~0.150	---
	Social participation	1.814 ***	0.262	0.020~0.515	14.4%
	Social support → social participation	1.814 ***	0.043	0.010~0.089	2.4%
High level	Social support	1.511 ***	0.086	−0.022~0.223	---
	Social participation	1.511 ***	0.480	0.224~0.761	31.8%
	Social support → social participation	1.511 ***	0.070	0.027~0.129	4.6%

ref = reference; c_i_ = the relative total effects of every category in categorical variables on the dependent variable; a_i_b = the quantity of relative mediation effect; |a_i_b/c_i_| = the proportion of relative mediation effect; CI = confidence interval. ** *p* < 0.01, *** *p* < 0.001 adjustment for age, marital status, smoking, drinking, nutrition status, and sleep quality.

## Data Availability

Data are available from the corresponding author upon reasonable request.

## Supplementary Materials

The following supporting information can be downloaded at https://www.mdpi.com/article/10.3390/healthcare13050551/s1, Table S1. Parallel mediating effect of social participation and social support on association of socioeconomic factors; Table S2. Serial mediating effect of social support through social participation on association of socioeconomic factors.
