# Supplementary material for: The Impact of Socioeconomic Factors on Cognitive Ability in Community-Dwelling Older Adults: Mediating Effect of Social Participation and Social Support
Source: Healthcare (Basel). 2025 Mar 4;13(5):551. doi: 10.3390/healthcare13050551 (PMC11899233; doi:10.3390/healthcare13050551)
Supplement: Supplementary file 1 [file healthcare-13-00551-s001.zip › healthcare-3487306-supplementary.pdf]

Supplementary Table S1. Parallel mediating effect of social participation and social support on association of socioeconomic factors

Relative direct effect of social participation medicated income on cognitive function

Path	β	S.E	t	P	LLCI	ULCI
Ref: low income						
Medium income→social participation	0.931	0.319	2.919	0.0036	0.305	1.556
High income→social participation	2.031	0.540	3.745	0.0002	0.962	3.080
Medium income→cognitive function	1.591	0.391	4.068	0.0001	0.824	2.359
High income→cognitive function	3.022	0.664	4.550	<0.001	1.719	4.325
Social participation→cognitive function	0.385	0.039	9.794	<0.001	0.308	0.462

Relative indirect effects of social participation medicated income on cognitive function

Path	Effect	S.E	Bootstrap CI 95%
Relative indirect effects (ref: low income)			
Medium income→social participation→cognitive function	0.358	0.129	0.110~0.614
High income→social participation→cognitive function	0.777	0.224	0.349~1.222
Relative direct effect (ref: low income)			
Medium income→cognitive function	1.591	0.391	0.824~2.359
High income→cognitive function	3.022	0.664	1.719~4.325
Relative total effects			
Medium income→cognitive function	1.949	0.408	1.148~2.750
High income→cognitive function	3.799	0.691	2.443~5.155

Relative direct effect of social support medicated income on cognitive function

Path	β	S.E	t	P	LLCI	ULCI
Ref: low income						
Medium income→social support	4.610	1.144	4.029	<0.001	2.365	6.855
High income→social support	5.605	1.937	2.894	0.004	1.805	9.406
Medium income→cognitive function	1.817	0.410	4.428	<0.001	1.012	2.623
High income→cognitive function	3.639	0.692	5.258	<0.001	2.281	4.997
Social support →cognitive function	0.029	0.011	2.500	0.013	0.006	0.051

Indirect effects of social support medicated income on cognitive function

Path	Effect	S.E	Bootstrap CI 95%
Relative indirect effects (ref: low income)			
Medium income→social support →cognitive function	0.132	0.065	0.019~0.282
High income→social support →cognitive function	0.160	0.089	0.017~0.372
Relative direct effect (ref: low income)			
Medium income→cognitive function	1.817	0.410	1.012~2.623
High income→cognitive function	3.639	0.692	2.281~4.997
Relative total effects			
Medium income→cognitive function	1.949	0.408	1.148~2.750
High income→cognitive function	3.799	0.691	2.443~5.155

Relative direct effect of social participation medicated occupation on cognitive function

Path	β	S.E	t	P	LLCI	ULCI
Ref: low occupation						
Medium occupation →social participation	0.910	0.310	2.939	0.003	0.303	1.518
High occupation →social participation	1.425	0.314	4.544	<0.001	0.810	2.041
Medium occupation →cognitive function	0.904	0.384	2.355	0.019	0.151	1.658
High occupation →cognitive function	1.014	0.391	2.591	0.010	0.246	1.782
Social participation →cognitive function	0.393	0.040	9.922	<0.001	0.316	0.471

Indirect effects of social participation medicated occupation on cognitive function

Path	Effect	S.E	Bootstrap CI 95%
Relative indirect effects (ref: low occupation)			
Medium occupation→social participation →cognitive function	0.358	0.125	0.123~0.614
High occupation→social participation →cognitive function	0.561	0.139	0.299~0.851
Relative direct effect (ref: low occupation)			
Medium occupation →cognitive function	0.904	0.384	0.151~1.658
High occupation →cognitive function	1.014	0.391	0.246~1.782
Relative total effects			
Medium occupation →cognitive function	1.262	0.401	0.475~2.049
High occupation →cognitive function	1.574	0.406	0.777~2.372

Relative direct effect of social support medicated occupation on cognitive function

Path	β	S.E	t	P	LLCI	ULCI
Ref: low occupation						
Medium occupation→social support	0.313	1.123	0.278	0.781	-1.891	2.516
High occupation→social support	0.956	1.137	0.841	0.401	-1.276	3.188
Medium occupation→cognitive function	1.251	0.399	3.134	0.002	0.468	2.034
High occupation→cognitive function	1.540	0.405	3.808	<0.001	0.747	2.334
Social support →cognitive function	0.036	0.011	3.119	0.002	0.013	0.058

Indirect effects of social support medicated occupation on cognitive function

Path	Effect	S.E	Bootstrap CI 95%
Relative indirect effects (ref: low occupation)			
Medium occupation→social support →cognitive function	0.011	0.043	-0.073~0.103
High occupation→social support →cognitive function	0.034	0.045	-0.042~0.132
Relative direct effect (ref: low occupation)			
Medium occupation →cognitive function	1.251	0.399	0.468~2.034
High occupation →cognitive function	1.540	0.405	0.747~2.334
Relative total effects			
Medium occupation →cognitive function	1.262	0.401	0.475~2.049
High occupation →cognitive function	1.574	0.406	0.777~2.372

Direct effects of social participation medicated education on cognitive function

Path	β	S.E	t	P	LLCI	ULCI
Ref: low education						
Medium education→social participation	0.788	0.317	2.485	0.013	0.166	1.410
High education→social participation	1.423	0.317	4.487	<0.001	0.800	2.045
Medium education→cognitive function	1.503	0.391	3.841	<0.001	0.735	2.271
High education→cognitive function	0.949	0.394	2.408	0.016	0.175	1.722
Social participation →cognitive function	0.395	0.040	9.978	<0.001	0.317	0.473

Indirect effects of social participation medicated education on cognitive function

Path	Effect	S.E	Bootstrap CI 95%
Relative indirect effects (ref: low education)			
Medium education→social participation →cognitive function	0.311	0.128	0.058~0.572
High education→social participation →cognitive function	0.562	0.140	0.294~0.853
Relative direct effect (ref: low education)			
Medium education →cognitive function	1.503	0.391	0.735~2.271
High education →cognitive function	0.949	0.394	0.175~1.722
Relative total effects			
Medium education →cognitive function	1.814	0.409	1.011~2.618
High education →cognitive function	1.511	0.409	0.707~2.314

Direct effects of social support medicated education on cognitive function

Path	β	S.E	t	P	LLCI	ULCI
Ref: low education						
Medium education→social support	3.108	1.138	2.732	0.006	0.875	5.341
High education→social support	5.043	1.138	4.431	<0.001	2.810	7.276
Medium education→cognitive function	1.718	0.410	4.193	<0.001	0.914	2.522
High education→cognitive function	1.355	0.412	3.286	0.001	0.546	2.164
Social support →cognitive function	0.031	0.012	2.679	0.008	0.008	0.054

Indirect effects of social support medicated education on cognitive function

Path	Effect	S.E	Bootstrap CI 95%
Relative indirect effects (ref: low education)			
Medium education→social support →cognitive function	0.096	0.054	0.013~0.214
High education→social support →cognitive function	0.156	0.075	0.035~0.318
Relative direct effect (ref: low education)			
Medium education →cognitive function	1.718	0.410	0.914~2.522
High education →cognitive function	1.355	0.412	0.546~2.164
Relative total effects			
Medium education →cognitive function	1.814	0.409	1.011~2.618
High education →cognitive function	1.511	0.409	0.707~2.314

Ref=reference category, SE=standard error, CI=confidence interval, *CI*=confidence interval, LLCI=lower level confidence interval, ULCI=upper level confidence interval

Supplementary Table S2. Serial mediating effect of social support through social participation on association of socioeconomic factors

Relative direct effect of social support through social participation medicated income on cognitive function

Path	β	S.E	t	P	LLCI	ULCI
Ref: low income						
Medium income →social support	4.610	1.144	4.029	<0.001	2.365	6.855
High income →social support	5.605	1.937	2.894	0.004	1.805	9.406
Medium income →social participation	0.760	0.319	2.384	0.017	0.134	1.385
High income →social participation	1.813	0.537	3.373	0.001	0.758	2.868
Social support →social participation	0.037	0.009	4.175	<0.001	0.020	0.055
Medium income →cognitive function	1.531	0.394	3.887	<0.001	0.758	2.303
High income →cognitive function	2.954	0.666	4.437	<0.001	1.647	4.261
Social support →cognitive function	0.015	0.011	1.321	0.184	-0.007	0.036
Social participation →cognitive function	0.378	0.04	9.535	<0.001	0.300	0.455

Relative indirect effects of social support through social participation medicated income on cognitive function

Path	Effect	S.E	Bootstrap CI 95%
Relative indirect effects (ref: low income)			
Medium income →social support →cognitive function	0.067	0.056	-0.032~0.193
High income →social support →cognitive function	0.082	0.072	-0.038~0.243
Medium income →social participation →cognitive function	0.287	0.125	0.042~0.539
High income→ social participation →cognitive function	0.685	0.221	0.261~1.136
Medium income →social support →social participation →cognitive function	0.065	0.023	0.025~0.115
High income →social support →social participation → cognitive function	0.078	0.033	0.023~0.152
Relative direct effect (ref: low income)			
Medium income →cognitive function	1.531	0.394	0.758~2.303
High income →cognitive function	2.954	0.666	1.647~4.261
Relative total effects (ref: low income)			
Medium income →cognitive function	1.949	0.408	1.148~2.750
High income →cognitive function	3.799	0.691	2.443~5.155

Relative direct effect of social support through social participation medicated occupation on cognitive function

Path	β	S.E	t	P	LLCI	ULCI
Ref: low occupation						
Medium occupation →social support	0.313	1.123	0.278	0.781	-1.891	2.516
High occupation →social support	0.956	1.137	0.841	0.401	-1.276	3.188
Medium occupation →social participation	0.898	0.307	2.928	0.003	0.296	1.499
High occupation →social participation	1.387	0.311	4.465	<0.001	0.777	1.997
Social support →social participation	0.040	0.009	4.559	<0.001	0.023	0.057
Medium occupation →cognitive function	0.907	0.383	2.366	0.018	0.155	1.660
High occupation →cognitive function	1.010	0.391	2.583	0.010	0.243	1.776
Social support →cognitive function	0.020	0.011	1.841	0.066	-0.001	0.042
Social participation →cognitive function	0.383	0.040	9.562	<0.001	0.304	0.461

Relative indirect effect of social support through social participation medicated occupation on cognitive function

Path	Effect	S.E	Bootstrap CI 95%
Relative indirect effects (ref: low occupation)			
Medium occupation →social support →cognitive function	0.006	0.027	-0.047~0.070
High occupation →social support →cognitive function	0.019	0.029	-0.028~0.090
Medium occupation →social participation →cognitive function	0.344	0.121	0.117~0.591
High occupation →social participation →cognitive function	0.531	0.139	0.277~0.824
Medium occupation →social support →social participation →cognitive function	0.005	0.018	-0.030~0.044
High occupation →social support →social participation →cognitive function	0.015	0.018	-0.019~0.055
Relative direct effect (ref: low occupation)			
Medium occupation →cognitive function	0.907	0.383	0.155~1.660
High occupation →cognitive function	1.010	0.391	0.243~1.776
Relative total effects (ref: low occupation)			
Medium occupation →cognitive function	1.262	0.401	0.475~2.049
High occupation →cognitive function	1.574	0.406	0.777~2.372

Relative direct effect of social support through social participation medicated education on cognitive function

Path	β	S.E	t	P	LLCI	ULCI
Ref: low education						
Medium education →social support	3.108	1.138	2.732	0.006	0.875	5.341
High education →social support	5.043	1.138	4.431	<0.001	2.810	7.276
Medium education →social participation	0.676	0.316	2.141	0.033	0.056	1.296
High education →social participation	1.241	0.318	3.906	<0.001	0.618	1.865
Social support →social participation	0.036	0.009	4.044	<0.001	0.019	0.053
Medium education →cognitive function	1.456	0.392	3.713	<0.001	0.687	2.226
High education →cognitive function	0.874	0.397	2.203	0.028	0.096	1.653
Social support →cognitive function	0.017	0.011	1.529	0.127	-0.005	0.039
Social participation →cognitive function	0.387	0.040	9.704	<0.001	0.309	0.465

Relative indirect effect of social support through social participation medicated education on cognitive function

Path	Effect	S.E	Bootstrap CI 95%
Relative indirect effects (ref: low education)			
Medium education →social support →cognitive function	0.053	0.042	-0.013~0.150
High education →social support →cognitive function	0.086	0.063	-0.022~0.223
Medium education →social participation →cognitive function	0.262	0.126	0.020~0.515
High education →social participation →cognitive function	0.480	0.137	0.224~0.761
Medium education →social support →social participation →cognitive function	0.043	0.020	0.010~0.089
High education →social support →social participation →cognitive function	0.070	0.026	0.027~0.129
Relative direct effect (ref: low education)			
Medium education →cognitive function	1.454	0.392	0.687~2.226
High education →cognitive function	0.874	0.397	0.096~1.653
Relative total effects (ref: low education)			
Medium education →cognitive function	1.814	0.409	1.011~2.618
High education →cognitive function	1.511	0.409	0.707~2.314

Ref=reference category, SE=standard error, CI=confidence interval, *CI*=confidence interval, LLCI=lower level confidence interval, ULCI=upper level confidence interval
